# Ultrasound your NPO: Effect of body mass index on gastric volume in term pregnant women – Retrospective case series

**DOI:** 10.1016/j.amsu.2019.10.029

**Published:** 2019-11-06

**Authors:** Efrain Riveros-Perez, Sherwin Davoud, Maria Gabriela Sanchez, Hugo Montesinos, Alexander Rocuts

**Affiliations:** a Department of Anesthesiology and Perioperative Medicine, Medical College of Georgia at Augusta University, 1120 15th Street, Augusta, GA, USA; b The Outcomes Research Consortium, Cleveland Clinic, Cleveland, OH, USA; c Medical College of Georgia, USA; d Department of Anesthesiology and Perioperative Medicine, Medical College of Georgia at Augusta University, USA; e Department of Mathematics and Computer Science, Ursinus College, Collegeville,PA, USA

**Keywords:** Ultrasound, Antral area, Pregnancy, Gastric volume, Preoperative fasting

## Abstract

**Introduction:**

A common belief has been that obese patients are prone to develop aspiration of gastric contents when general anesthesia is administered. We aimed to determine the correlation between antral cross-sectional area as a surrogate of gastric volume measured by gastric ultrasound, and body mass index (BMI) in term pregnant women scheduled for elective cesarean section.

**Methods:**

A cross-sectional observational study was conducted on forty-two term pregnant patients scheduled for cesarean section. A preoperative qualitative and quantitative ultrasound assessment of the antral area was performed on the day of surgery. Gastric volume as a function of BMI was evaluated.

**Results:**

A significant correlation was found between BMI and gastric antral area (p = 0.001), as well as with longitudinal diameter (p < 0.001). This correlation is independent of gravidity and parity.

**Conclusion:**

BMI is an independent predictor of the antral cross-sectional area and gastric volume in term pregnant patients scheduled for cesarean section. Perioperative fasting guidelines in pregnancy should be adjusted in obese and morbidly obese pregnant women.

## Highlights

• Body mass index predicts gastric volume in term pregnant patients.
• The effect of body mass index on gastric volume is independent of gravidity and parity.
• Fasting guidelines in obstetrics should take into account body mass index.

## Teaser

**Synopsis:** Body mass index is a predictor of antral cross sectional area and gastric volume in pregnant patients undergoing cesarean section.

## 1 Introduction

Obesity in pregnancy is a growing problem in the developed world, affecting approximately 35% of adult women in the United States [1]. Anesthetic management of the obstetric patient with obesity and morbid obesity is challenging. A common belief has been that obese patients are prone to develop aspiration of gastric contents when general anesthesia is administered, due to increased intra-abdominal pressure and a higher incidence of hiatal hernia compared to non-obese individuals [2,3]. In addition, pregnancy confers risk due to physiologic changes mediated by progesterone, anatomical changes induced by the gravid uterus, and changes in gastric pH [4]. The risk is further increased during labor, especially when opioid medications are administered. Prophylaxis for gastric aspiration is advocated prior to general anesthesia in this patient population [5]. The obese and morbidly obese pregnant patients are particularly vulnerable to adverse outcomes related to bronchoaspiration.

Gastric volume and pH are the main variables determining risk and severity of aspiration [6]. Estimation of gastric volume with ultrasound has been proposed to supplement fasting guidelines in order to improve the quality of risk assessment in urgent situations and in patient populations at high risk of aspiration [7]. Ultrasonography has been previously used to assess gastric volume in a highly reproducible manner by clinical anesthesiologists at the bedside [8]. Correlation between gastric volume determined by ultrasound and that measured by gastric suctioning has been found in severely obese non-pregnant patients [9]. Pregnancy, on the other hand, is associated with significant intra-abdominal anatomical changes that may lead to stomach compression, making gastric volume estimation particularly challenging, especially in the context of obesity [10]. The combination of obesity and pregnancy has effects on gastric volume and its measurement.

This study is aimed to determine the correlation between antral cross-section as a surrogate of gastric volume measured by gastric ultrasound, and body mass index (BMI) in term pregnant women scheduled for elective cesarean section. We also aim to quantify the gastric volume measured with ultrasound as a function of fasting time in this patient population.

## 2 Methods

**Fig. 1:**
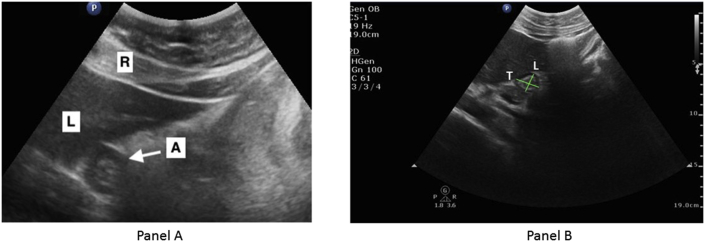
Transverse ultrasonographic view of gastric antrum (A arrow) in relation to the liver (L) and rectus abdominis muscle (R) (Panel A). Cross-sectional antral diameters.L, longitudinal cranio-caudal axis.T, transversal axis (Panel B).

A prospective consecutive case series study was conducted. The series was prospective, consecutive and conducted in a single center. After approval by the Institutional Board Review of Augusta University (expedited review), forty-two pregnant patients at term scheduled for elective cesarean section at a tertiary academic institution (with fasting times greater than 6 h) were invited to participate in this study. The study was registered in the Clinicaltrials.gov registry (NCT03555604). The paper is reported following the PROCESS guidelines criteria [11]. Patients with gastrointestinal disorders, taking pro-kinetic medications, having nausea or vomiting, and those with systemic diseases affecting gastric emptying (i.e. diabetes mellitus) were excluded. Participants signified their voluntary intent to participate by signing a university-approved informed consent document. Gastric ultrasound with a low frequency (1–5 Hz) curvilinear array transducer using a Philips (CX-50) (Bothell, WA. USA) with image compounding technology was performed in each patient. The ultrasound procedure was performed in the supine position and in a semi-recumbent right lateral position. The antrum was identified in the sagittal plane between the liver, pancreas and aorta, and between peristaltic contractions. Experienced operators (E.R.P. and A.R.) performed the ultrasound examinations. In order to guarantee quality control in terms of ultrasound quality and inter-observer variability, the two operators obtained ultrasound images in a pilot test before the initiation of the study, and the measurements were evaluated independently by a third party with experience in abdominal ultrasound. Qualitative assessment consisting of three grades was made [9]: Grade 1, no fluid evidenced; Grade 2, clear fluid only evidenced in right lateral decubitus position, and Grade 3, fluid evidenced in both positions. Quantitative measurement of the cross-sectional area of the antrum (CSA) was done by means of free tracing calipers. The full-thickness of the gastric wall was included in the measurement. CSA was calculated using the following formula [10]: CSA = (π x mean anteroposterior diameter x mean longitudinal diameter)/4 (Fig. 1). In addition to qualitative and quantitative ultrasound measures, we recorded demographic variables (age, BMI) and obstetric variables (weeks of gestation, gravity, parity) as well as fasting time.

### 2.1 Statistical analysis

Based on previous studies, we assumed a mean (SD) of 3 (1.5) cm^2^ for CSA. We wanted to detect a difference of 100 mm^2^ with a significance level of 0.05 and power of 80%. With this information, a sample size of 40 patients was deemed sufficient. We used descriptive statistical methods to analyze demographic characteristics of the population. All statistical analysis was performed using SPSS 20.0(IBM, Armonk, USA). We performed multiple regression analysis to determine the association between different predictors and the outcome variable CSA. Predictors were considered to be correlated when a p-value < 0.05 was obtained in relation to the z-statistic. Pearson analysis was performed to correlate BMI with other predictors (e.g. gestational age) and with gastric diameters.

## 3 Results

We enrolled 42 patients in the study. Demographic variables are shown in Table 1. Table 2 exhibits the summary measures for the variables of interest in our sample. The Pearson correlation coefficient between BMI and gestational age was 0.2017 (N = 42, p = 0.1649), and between BMI and the longitudinal diameter was 0.2799 (N = 42, p = 0.0895). One of our patients exhibited high residuals and high leverage. It is believed that this patient was not adequately fasted resulting in incorrect measurements for the reported *nil per os* (NPO) status (18 hours). We therefore present results with and without this case. The Pearson correlation coefficient between BMI and gestational age excluding this patient was 0.2519 (n = 41, p = 0.083) and between BMI and the longitudinal diameter was 0.3438 (N = 41, p = 0.035). The reported p-values, in all cases, are robust to heteroskedasticity. This means that even the inclusion of this patient does not affect the significance of our findings. Table 3 and subsequent analyses were done with the whole sample of forty-two patients.

**Table 1:** Demographic variables. SD, standard deviation. Long. External, cranio-caudal diameter. Short External, transversal antral diameter.

Variable (n = 42)	Mean	SD	Min	Max
BMI	36.07	8.12	24.34	65.4
Age	29	4.93	20	41
Gravidity	3	1.31	1	6
Parity	1.31	0.87	0	4
Gestational age (weeks)	38.52	0.85	36	40
Long. External (cm)	2.43	0.61	0.74	3.64
Short. External (cm)	1.56	0.37	0.86	2.29
External CSA (cm^2^)	2.99	1.07	0.79	5.00
NPO Status (hours)	11.75	3.15	4.5	19
Weight (Kg)	96.55	22.94	65	189
Height (cm)	163.43	6.56	148	177.8

**Table 2:** Gastric ultrasound variables. SD, standard deviation.

Variable (n = 42)	Mean	SD	Min	Max
Long External Axis (cm)	2.43	0.61	0.74	3.64
Short External Axis (cm)	1.56	0.37	0.86	2.29
External CSA (cm^2^)	2.99	1.07	0.79	5.00

**Table 3:** Multiple regression model for ultrasound measures as response variables (P- values in parentheses. *p < 0.1, **p < 0.05, ***p < 0.01).

	[1]	[2]	[3]	[4]
	External CSA	Long. External	External CSA	Long. External
BMI	0.0380**	0.0277**	0.0518***	0.0367***
	(0.046)	(0.022)	(0.001)	(0.000)

Gravida	0.326**	0.189**	0.426***	0.255***
	(0.018)	(0.035)	(0.000)	(0.000)

Intercept	0.642	0.860	−0.224	0.294
	(0.525)	(0.217)	(0.742)	(0.555)
*N*	42	42	41	41

Considering that variations in age, gravidity, parity, gestational age, NPO status, weight, and height may have influence over CSA, we performed multivariate regression analysis. The results are shown in Table 3. We evidenced a positive and significant relationship between BMI and CSA, and between BMI and the longitudinal diameter. Since both variables affect the gastric volume, our evidence indicates that BMI influences the gastric volume through the cross-sectional area of the antrum or, specifically, through the longitudinal diameter. The influence of BMI was larger in columns [3,4] of Table 3, where the patient with high residuals and leverage was excluded. All current models have variance inflation factors (VIF) lower than 1.08 reflecting absence of multi-collinearity. Height and weight were omitted since the VIF would rise to 77.80, indicating a high degree of multicollinearity with BMI. As before, all p-values were robust to heteroscedasticity.

The results are not only statistically significant, but they are large in magnitude. In the sample of 42 patients (columns 1 and 2), a one standard deviation increase in BMI is associated with increases of 0.288 standard deviations in CSA (p = 0.046), and 0.367 standard deviations in the longitudinal diameter (p = 0.022). In the sample of 41 patients (columns 3 and 4), one standard deviation increase in BMI was associated with 0.393 standard deviations increase in CSA (p = 0.001), and 0.494 standard deviations increase in the longitudinal diameter (p = 0.000).

## 4 Discussion

Our study shows that the BMI is positively related to antral gastric cross-sectional area and cranio-caudal longitudinal diameter, as surrogates of gastric volume in healthy term pregnant patients scheduled for elective cesarean section. In addition, the association is large in magnitude and is independent of gravidity and parity.

The risk of aspiration of gastric contents in the obstetric population was identified several decades ago as a significant source of poor outcomes [6,11]. In spite of the widespread use of regional anesthesia for cesarean section, bronchoaspiration remains an important concern and a significant cause of morbidity when general anesthesia needs to be administered [12,13]. Practitioners have assumed the association between “full stomach” and risk of aspiration based on physiological changes that affect pregnancy. These changes include the smooth muscle relaxation induced by progesterone and anatomical changes of intra-abdominal contents. On the other hand, the notion of increased gastric volume at full-term pregnancy has been challenged in recent years. The introduction of ultrasound technology to the obstetric anesthesia practice has permitted the evaluation of gastric volumes [14]. Barboni et al. showed that the transit of food after a meal is completed later in comparison to non-pregnant women [15]. Our findings agree with those of other authors showing that following currently accepted fasting guidelines is no guarantee for an empty stomach. Hakak et al. evidenced that after fasting 6 h, full-term parturients had quantitative ultrasonographic evidence of gastric volumes large enough to put patients at risk for aspiration [16]. In contrast, Van de Putte et al. found that term pregnant patients have similar gastric volume compared to non-pregnant women, assessed by qualitative and quantitative ultrasound measures [17].

Obesity has been identified as a risk factor for aspiration in the general population. However, there is controversy regarding the factors predisposing patients to this risk. Jackson et al. showed a significant delay in gastric emptying by noninvasive methods in non-pregnant women [18]. Altered motility in obese patients has been related to biochemical pathways that involve abnormal production of nitric oxide synthase [19]. In contrast, Buchholz et al. compared nineteen obese patients (BMI >40Kg/m^2^) with non-obese subjects by scintigraphy after a labeled meal, finding no differences in gastric emptying between the groups [20]. Other authors agree with this finding, attributing anesthetic risks to other factors such as hiatal hernia and anatomical variation in the obese population [20,21]. The combination of pregnancy and obesity has been postulated as a heightened risk factor for aspiration, mainly due to anatomical changes secondary to an enlarged uterus [3]. We may argue that BMI confers additional risk of aspiration by virtue of its effect on gastric volume (measured by its area surrogate on ultrasound). Although current perioperative fasting guidelines mention pregnancy and obesity as separate risk factors for aspiration, our study provides insights into the notion that BMI in pregnancy exaggerates the risk, and that the magnitude of that risk is higher at higher levels of BMI.

The Practice Guidelines for Obstetric Anesthesia issued by the Task Force of the American Society of Anesthesiologists on Obstetric Anesthesia and the Society for Obstetric Anesthesia and Perinatology acknowledge that the coexistence of pregnancy and morbid obesity may warrant stricter restrictions of oral intake [22]. Our results show that one size recommendation does not fit all patients. Pregnant women with higher BMI exhibited increased CSA (and gastric volume), arguably putting them at a higher risk for aspiration compared to pregnant patients with lower BMI. We may argue that BMI as a variable should be factored in before making a decision in relation to fasting status of patients scheduled for cesarean section. Our study adds to the current knowledge, as we are showing that BMI constitutes an independent variable to predict gastric volume as a possible risk factor for gastric aspiration in obstetric patients. We strongly recommend that BMI be included in the decision tree in relation to preoperative fasting guidelines for scheduled cesarean section.

Our study has limitations. The quality of ultrasound exam is highly dependent on operator's experience. In our study, only two anesthesiologists performed the studies, which limits the application of our results to contexts where providers are at earlier stages in the learning curve. The study was conducted in healthy pregnant patients at term scheduled for cesarean section. The results cannot be extrapolated to other relevant situations such as labor, emergent procedures, and other gestational ages. Ultrasonography has inherent limitations including the need for a good window in order to produce a high-quality image suitable for interpretation. Additionally, presence of gastric gas may limit the quality of the study in some circumstances. Further research is necessary to define the clinical role of gastric ultrasound as a diagnostic tool for gastric volume in the perioperative period of pregnant patients. It is important to note that our study used gastric CSA, and is not measuring gastric volume directly. Although correlation between both measurements has been reported, geometric variations of gastric fundus and orientation may affect this correlation [23]. A multicenter study measuring gastric volume in patients with diverse demographic characteristics and backgrounds would strengthen the evidence to support changes in fasting guidelines.

In conclusion, the BMI is an independent predictor of antral cross sectional area and gastric volume in term pregnant patients scheduled for cesarean section. Perioperative fasting guidelines in pregnancy should be adjusted in obese and morbidly obese pregnant women.

## Funding

This study was funded by the Department of Anesthesiology and Perioperative Medicine of Augusta University.

## Provenance and peer review

Not commissioned, externally peer reviewed.

## Ethical approval

IRB approval (Augusta University). Project number: **1184342.**

## Sources of funding

None.

## Author contribution

Efrain Riveros-Perez: Data collection and analysis. Manuscript construction.

Sherwin Davoud: Data collection and analysis. Manuscript review.

Maria Gabriela Sanchez: Data collection. Manuscript construction.

Hugo Montesinos: Data collection analysis. Manuscript construction.

Alexander Rocuts: Data collection and analysis. Manuscript review.

## Research registry number

N/A.

## Guarantor

Efrain Riveros-Perez.

## Declaration of competing interest

None.
